# A monster into the heart: an unusual presentation of cardiac leiomyosarcoma

**DOI:** 10.1186/s43044-023-00429-3

**Published:** 2023-12-19

**Authors:** Raul Cruz Palomera, Jose Dario Valencia Gonzalez, Juan Guzmán Olea, Rosa Elena Gutiérrez Castañeda, Juan Francisco Rodríguez Alvarado, Juan Camacho Huembes, Jorge Guillermo Arenas Fonseca, Alejandro Carcaño Cuevas, Gabriel Guzman Olea

**Affiliations:** Department of Cardiology, Instituto Mexicano Del Seguro Social, 2 Norte 2004 Col Centro, Puebla, Puebla Mexico

**Keywords:** Heart neoplasms, Leiomyosarcoma, Primary cardiac tumors, Sarcoma, Case report

## Abstract

**Background:**

Malignant primary cardiac tumors are infrequent and can lead to an unfavorable prognosis if not identified and treated promptly. Early detection and prompt treatment of malignant primary cardiac tumors are crucial for a better prognosis. This article presents a case of primary cardiac leiomyosarcoma and reviews the literature on this topic.

**Case presentation:**

Female patient that developed recurrent pericardial effusion and hemodynamic instability caused by a cardiac tumor, later identified as leiomyosarcoma. Multidisciplinary treatment was administered to the patient.

**Conclusions:**

The initial approach to this type of pathology should include multimodality imaging to establish a prompt diagnosis leading to complete standard treatment, to minimize risks to the patient's heart function which may include resection with complete margins of the neoplasm, otherwise the prognosis may be poor.

## Background

Cardiac tumors are rare and consist of a variety of neoplastic or non-neoplastic lesions. When neoplastic cardiac tumors are present, they are classified into primary tumors (PCT) or secondary "metastases." It is worth nothing that up to 90% of PCTs are benign, where the most dominant histological subtype is myxoma. In contrast, malignant PCTs are rare, accounting for only 5–6% [1].

Among the malignant PCTs, sarcomas are the most common at (64.8%), followed by lymphomas (27%) and mesotheliomas (8%) [2]. Leiomyosarcomas account for less than 1% of all malignant PCTs and make up only 8–9% of cardiac sarcomas [3].

The clinical presentation of PCTs varies, with some patients exhibiting symptoms, while others are asymptomatic. Asymptomatic patients may detect the tumor incidentally during another pathology evaluation. The classic triad of PCTs includes symptoms and signs due to intracardiac obstruction, systemic embolization signs, and systemic or constitutional symptoms. Dyspnea and orthopnea resulting from pulmonary venous hypertension or pulmonary edema are frequently observed cardiac symptoms [4]. The PCTs are diagnosed through transthoracic and transesophageal echocardiograms, magnetic resonance imaging (MRI), and computed tomography (CT) scans [4]. Tumor resection is considered the preferred treatment option [5].

Primary cardiac leiomyosarcoma is a rare yet highly aggressive malignant mesenchymal neoplasm comprised cells that show structural or antigenic proof of smooth muscle differentiation. Patients with leiomyosarcoma have a mean age of 45 years (with a range from 6 weeks to 77 years), and the incidence of this condition is twice as common in females [6]. The left atrium is the most common site of origin, usually presenting as sessile masses with a mucoid appearance. These tumors typically grow outwardly and have a high incidence of lungs metastasis.

The clinical presentation varies according to tumor location and size. Usually, there are no symptoms until an advanced stage is reached, and diagnosis is commonly delayed until clinical symptoms manifest, such as heart failure and embolism [7].

Transthoracic echocardiogram is the primary imaging method for demonstrating tumor size, extension, mobility, location, and proximity to adjacent structures. Transesophageal echocardiogram is more useful in tumors less than 5 mm and those located in the posterior wall [8]. These tumors have a poor prognosis, with a reported mean survival time of 6 months from the time of diagnosis if left untreated [9]. Surgical resection is primarily palliative. The role of adjuvant radiotherapy and/or chemotherapy remains a topic of controversy [6].

The current case belongs to an exceptional occurrence of primary cardiac leiomyosarcoma that affected the anterior wall of the trunk of the pulmonary artery, leading to an unusual presentation of cardiac tamponade.

## Case presentation

A 69-year-old woman, without cardiovascular risk factors or cardiovascular history. She arrived at hospital with functional class NYHA II and postprandial fullness. Physical examination findings were not significant.

**Fig. 1:**
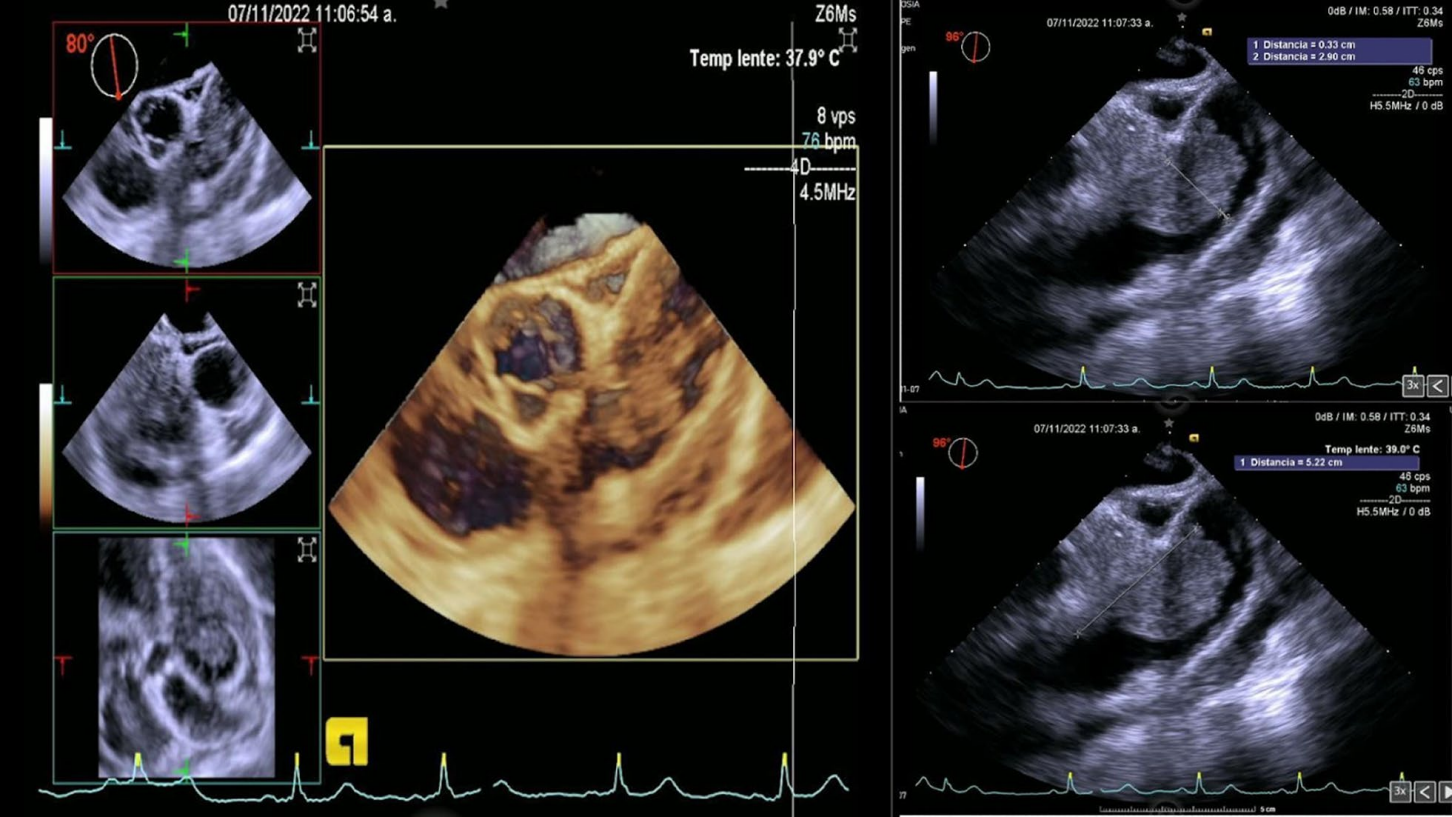
Transesophageal echocardiogram. A tumor mass fixed to the anterior wall of the trunk of the pulmonary artery

**Fig. 2:**
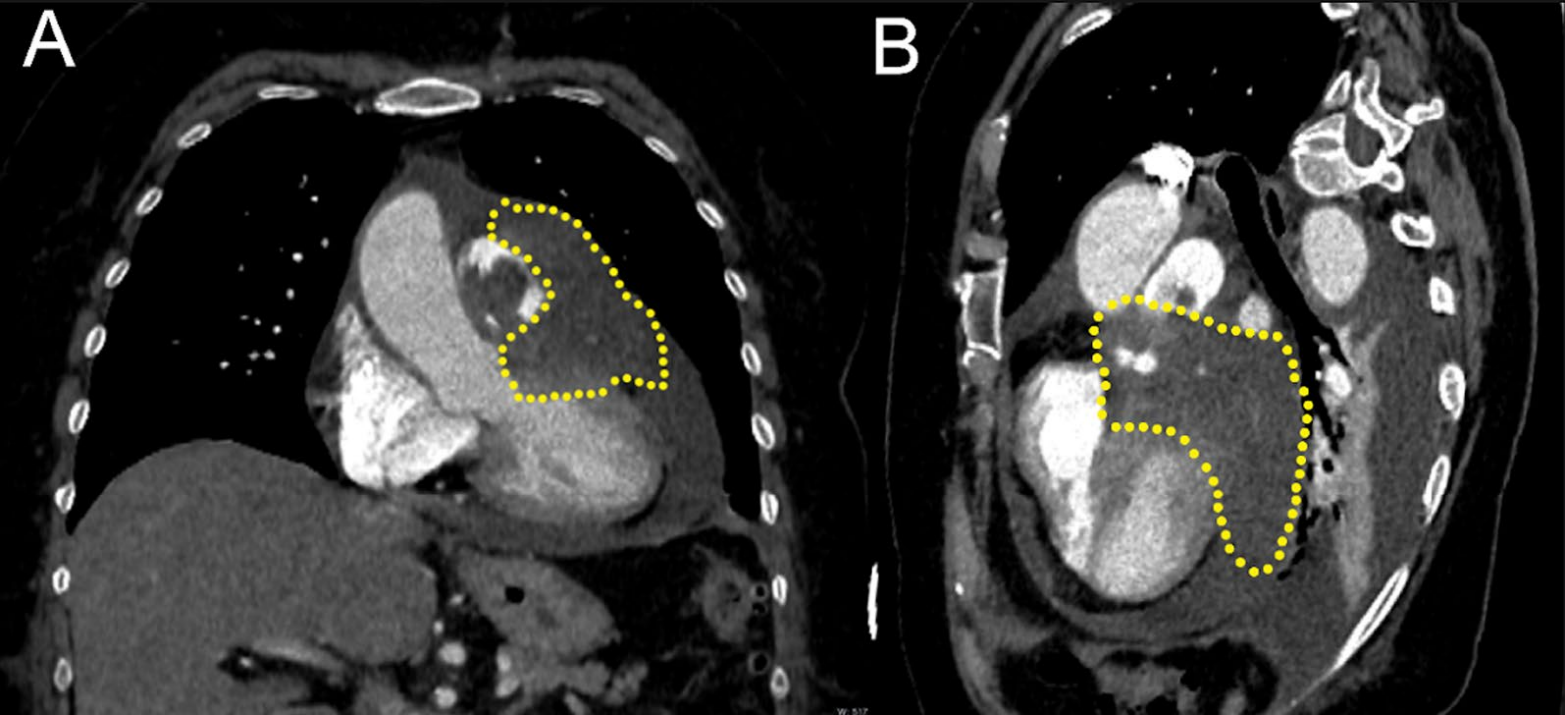
Chest tomography. **A**, **B** A Coronal and oblique views on a CT, confirming the location of the cardiac tumor

The diagnostic protocol commenced with a chest X-ray revealing cardiomegaly. Subsequently, point-of-care ultrasound (POCUS) detected pericardial effusion along with hemodynamic compromise, necessitating pericardiocentesis that led to partial clinical improvement. Nonetheless, the patient experienced clinical deterioration again, requiring referral to our medical center. A transthoracic and transesophageal echocardiogram detected right ventricular systolic dysfunction, pericardial effusion with 26 mm separation between sac leaves in the left ventricular free wall, and a tumor mass fixed to the anterior wall of the pulmonary artery trunk (orthogonal diameter 29 × 52 mm) with multilobed characteristics, the mass exhibited areas of heterogeneous density and hypogenicity, which were suggestive of necrosis. Color doppler flow detected in the mass suggested vascularization, leading to a 90% obstruction of the main lumen of the pulmonary artery with an obstructive gradient of 68 mmHg. At the proximal end of the mass, it infiltrated the free and anterior wall of the left ventricle, and collapse of the left atrial appendage (Fig. 1). The imaging protocol using chest tomography was conducted and confirmed the lesion's location. It was described as a lobulated exophytic mass with low attenuation and peripheral enhancement, accompanied by pleural effusion and thrombi in the pulmonary artery trunk (Fig. 2), provides visual representation of these findings.

**Fig.3:**
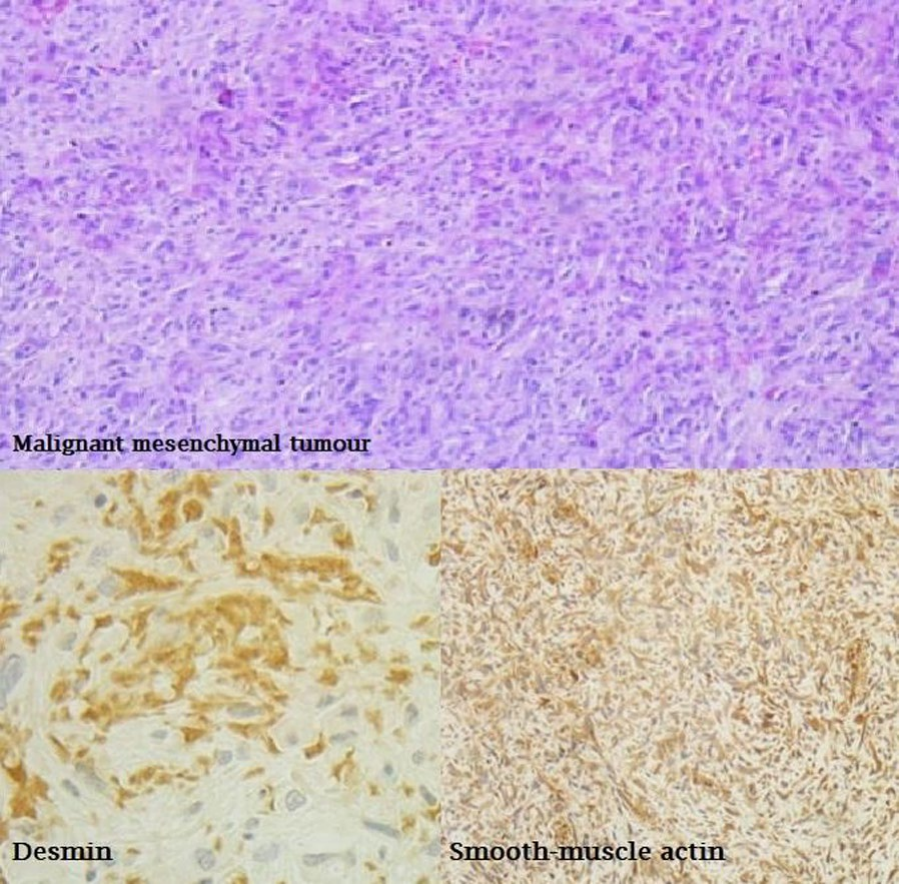
Biopsy of the tumor. A primary cardiac leiomyosarcoma with positive immunohistochemistry for smooth muscle actin and desmin

The patient's case was evaluated by the heart team, and it was concluded that due to the recurrence of the pericardial effusion and findings, a pericardial window with a biopsy of the tumor was necessary. The tumor biopsy report confirmed the presence of a primary cardiac leiomyosarcoma with positive immunohistochemical results for smooth muscle actin and desmin (Fig. 3). The patient received an oncology evaluation that showed evidence of metastatic tumor activity in the brain magnetic resonance imaging; therefore, only palliative treatment was recommended.

The patient experienced clinical decline related to ventricular dysfunction, resulting in death 5-month post-diagnosis.

## Discussion

Primary cardiac leiomyosarcoma is a rare condition that occurs in less than 0.25% of all cardiac tumors [7]. It is highly aggressive, with only 200 cases reported in the literature [10] and a higher incidence among females, as observed in this case. Patients affected by this condition typically have a lower age average than the present case. Generally, 3 types are described: cutaneous, soft tissue, and vascular. In this case, the neoplasm was located in the anterior wall of the pulmonary artery trunk, which is of particular interest because it is not commonly found in this location. The typical presentation site for this condition is the left atrium, accounting for up to 50% of cases.

During the preliminary examination, it is necessary to assess the clinical manifestation by examining certain symptoms based on their stage and location. These symptoms include chest pain, dyspnea, and non-productive cough, as well as data of right heart failure, valvular stenosis, rhythm disturbances, pericardial disease, or sudden death. When the presentation is associated with pericardial disease, it is important to consider cardiac tamponade (dyspnea, tachypnea, jugular venous distention, arterial hypotension, muffled heart sounds) as in the present case.

Similarly, determining the location within cardiac structures is essential, warranting the significance of adopting a multimodal cardiac imaging approach. Transthoracic echocardiography (TTE) is a readily accessible screening test and remains the gold standard for identifying cardiac tumors [11]. Furthermore, in this case, although cardiac tamponade is a clinical diagnosis, the echocardiogram allows for an assessment of the characteristics of the pericardial effusion and determination of any hemodynamic compromise. In these types of lesions, TTE determines the location, mobility, size, and pericardial condition of the tumor, while assessing the vascularity of the tumor, with increased vascularity suggesting malignancy, as in our case. Additionally, transesophageal modality could be employed as a complement to TTE, allowing for assessment of structural changes, precise determination of small structures, and identification of the origin and infiltration of the lesion. When used in combination with three-dimensional echocardiography, a more accurate evaluation of the anatomical relationships, size, and shape of the mass can be achieved, which is critical in establishing a diagnosis.

There are additional imaging studies in the presence of a cardiac mass with data suggestive of neoplasia. Magnetic resonance imaging is a valuable tool for assessing tumor volume, burden, mediastinal invasion, and response to therapy [12]. In this instance, the necessary study was unavailable, so we proceeded with a diagnostic approach that utilized chest tomography. Although this imaging technique is less commonly used, it is capable of assessing cardiac masses, particularly when other methods are either inconclusive or not viable. Confirmation of the diagnosis was obtained via pathological and immunohistochemical analysis [10]. Histologically, this tumor is characterized by compact bundles of spindle cells with blunt nuclei, regions of necrosis, and often mitotic figures with epithelioid regions [4].

The treatment of this entity should be multidisciplinary, with tumor resection with wide margins as the main objective, which increases survival. Surgical intervention is reserved for cases which present with hemodynamic compromise due to mass effect, increased risk of embolism, and the presence of cardiac arrhythmias. The effectiveness of treatment relies on the neoplasia stage, presence of metastases, and the patient's general condition. While tumor resection and chemotherapy combination may be effective, its actual efficacy remains unclear due to leiomyosarcoma's rarity [7]. The prognosis varies depending on the type of resection. For incomplete resections, the average survival is 6–12 months, whereas complete resections have a survival rate of 24–34 months.

## Conclusions

This neoplasm has a poor prognosis, and early diagnosis is crucial via clinical evaluation and multimodal cardiac imaging, confirmed by histopathology. The preferable treatment is complete surgical resection with wide margins, which enhances the patient's prognosis and survival.

## Data Availability

Not applicable.

## Abbreviations

PCT: Primary cardiac tumors
TTE: Transthoracic echocardiography
POCUS: Point-of-care ultrasound
NYHA: New York Heart Association
CT: Computed tomography
